# Blood heavy metal and trace element levels in newly diagnosed cases of inflammatory bowel disease

**DOI:** 10.12669/pjms.42.7.14755

**Published:** 2026-07

**Authors:** Enver Akbas, M. Salih Akın, Gozde Ulfer

**Affiliations:** 1 Enver Akbas, MD Department of Gastroenterology, Faculty of Medicine, Istanbul Medipol University, Istanbul, Turkey; 2 M. Salih Akın, MD Department of Gastroenterology, Faculty of Medicine, Istanbul Medipol University, Istanbul, Turkey; 3 Gozde Ulfer, MD Department of Biochemistry, Faculty of Medicine, Istanbul Medipol University, Istanbul, Turkey

**Keywords:** Crohn’s disease, Heavy metals, Trace elements, Ulcerative colitis

## Abstract

**Objectives:**

The relationship between heavy metal and trace element levels in IBD and the clinical course is unclear. The research team evaluated serum levels of heavy metals and trace elements in patients with IBD compared with healthy participants to establish their connection with IBD severity and inflammatory markers.

**Methodology:**

This single-center, retrospective cross-sectional study was conducted at the Department of Gastroenterology, Istanbul Medipol University Faculty of Medicine, Istanbul, Turkey, with stored samples analyzed from 17 October to 17 November 2024. The study included 137 patients with IBD (90 with ulcerative colitis, 47 with Crohn’s disease) and 60 healthy controls. Patients were grouped according to disease severity as mild, moderate, and severe. Serum levels of heavy metals (nickel, aluminum) and trace elements (iron, zinc, selenium, ferritin, vitamin D) were measured. Correlations with C-reactive protein (CRP), sedimentation rate, and fecal: calprotectin were examined.

**Results:**

Nickel levels were higher in the IBD group than in controls (1.19±1.04 vs 0.51±0.39 µg/L; median 1.2 [IQR 0.5-1.6] vs 0.4 [0.2-0.7] µg/L; p<0.001). Iron deficiency was present in 54% of patients compared with 16.7% of controls (p<0.001). Zinc and ferritin levels were significantly lower in the patient group (p<0.001). Aluminum strongly correlated with CRP (r=0.69, p<0.001). Multivariate analysis showed a 7.36-fold higher likelihood of IBD associated with elevated nickel levels (OR=7.36; 95% CI: 3.23–16.75; p<0.001).

**Conclusion:**

Nickel accumulation and iron deficiency were associated with IBD severity and may impact pathogenesis. No independent relationship was established for other heavy metals and micronutrients.

## INTRODUCTION

Inflammatory bowel diseases (IBD) are increasingly common, yet their causes remain unidentified, with environmental factors believed to be involved. Ingestion of lead, cadmium, arsenic, and manganese induces oxidative stress, increases intestinal permeability, causes epithelial dysfunction, and triggers proinflammatory cytokine release via gut microbiota imbalance, leading to chronic inflammation.^1^,^2^ Iron deficiency in ~50% of IBD cases and zinc and selenium deficiencies negatively affect disease course.^3^ Existing research on heavy metals and IBD primarily involves Western populations, rather than developing countries such as Turkey. We investigated serum concentrations of heavy metals and trace elements in patients with IBD and healthy participants to determine any possible involvement in IBD development.

## METHODOLOGY

This single-center, retrospective cross-sectional study was conducted at the Department of Gastroenterology, Istanbul Medipol University Faculty of Medicine, Istanbul, Turkey. IBD data collected between 2023 and 2025 were re-analysed after ethics approval, and stored samples were tested from 17 October to 17 November 2024. G*Power 3.1 was used to calculate that a sample size of 128 patients with IBD and 54 controls would be sufficient to detect an effect size of 0.5 with 80% power at a 5% significance level. The mild severity subgroup (n=10) reflects the natural distribution of newly diagnosed cases meeting the inclusion criteria during the study period; no minimum subgroup size was prespecified, and this limitation is acknowledged accordingly.

### Ethical approval:

Ethics committee approval for the retrospective use of these samples was subsequently granted by the Istanbul Medipol University Non-Interventional Clinical Research Ethics Committee (no. 1194, October 16, 2025). Written informed consent was obtained from all participants, and the study complied with the Declaration of Helsinki and Good Clinical Practice.

The study included 137 participants with IBD who had endoscopy-confirmed ulcerative colitis or Crohn’s disease and were at least 18 years old and 60 participants who were healthy controls with normal colonoscopy results and no chronic diseases or regular medication use. Exclusion criteria comprised heavy-metal exposure, cancer, pregnancy, infection or antibiotic use within three months.

Disease severity was graded using the Mayo score for ulcerative colitis (mild: 3–5; moderate: 6–10; severe: >10) and the Crohn’s Disease Activity Index (CDAI) for Crohn’s disease (remission: <150; mild: 150–220; moderate: 221–450; severe: >450).^4^ The endoscopic evaluation of ulcerative colitis identifies four subtypes which include proctitis and left-sided and extensive and pancolitis patterns.^5^

The laboratory collected morning fasting blood which they divided into two types of tubes containing citrate solution for metal analysis and serum solution for trace element analysis. The blood samples underwent centrifugation at 1500 × g for 10 minutes, followed by storage at −80°C for subsequent analysis which took place between 6–30 months later.^6^ The ICP-MS instrument (Agilent 7800, Agilent Technologies, Santa Clara, CA, USA) measured Pb, Cd, Hg, As, Ni, Al, and Sn levels in blood samples at µg/L concentrations. Although Pb, Cd, Hg, As, and Sn were also measured by ICP-MS, their concentrations remained below the limit of detection in the majority of samples and showed no statistically significant differences between groups; therefore, only nickel and aluminum data are reported in the Results and Discussion sections. The laboratory used colorimetric methods for serum iron and zinc, ICP-MS for selenium (µg/L), chemiluminescence for vitamin D (ng/mL), and electrochemiluminescence for ferritin (ng/mL).^6^ The researchers conducted CRP, ESR and fecal calprotectin tests for correlation analysis. All laboratory analyses were conducted at a single laboratory using calibrated equipment, with personnel blinded to group allocation. The study established the following measurement ranges: Se 70–150 µg/L,^7^ Fe 50–170 µg/dL,^8^ Ni 0–1 vs >1 µg/L,^9^ vitamin D <20 deficient, 20–30 insufficient, >30 sufficient,^10^ Al 0–6 vs >6 µg/L.^11^

### Statistical analyses:

Statistical analyses were conducted using SPSS 25.0. The Kolmogorov–Smirnov test evaluated normal distribution patterns in the data. As the majority of continuous variables showed non-normal distribution, results are presented as both mean ± SD and median [interquartile range, IQR] to ensure transparent representation of skewed distributions, particularly in subgroups with small sample sizes. Categorical data were shown as n (%). The research used Mann–Whitney U for two-group contrasts between IBD and control groups but Kruskal–Wallis with Dunn post-hoc for three or more group comparisons (subtype or severity). UC localizations (proctitis, left-sided, extensive, pancolitis) were compared similarly, with categorical variables analyzed using the χ² test. Spearman correlation linked metals with CRP, ESR and fecal calprotectin. Multivariate logistic regression included age and sex, together with Ni, Al, Se, vitamin D, Zn, and Fe that were significant in univariate analysis. ROC curves yielded AUC, cut-off, sensitivity, and specificity; only Ni reached an AUC of 0.70. Missing data were <2%, completeness was >98%, and all tests were two-tailed.

## RESULTS

The present study included 137 patients with IBD and 60 healthy individuals. The mean ages (control: 36.7; IBD: 36.4 years) and sex distribution were similar between the groups. The majority of patients had ulcerative colitis, and approximately half were in the moderate category while half belonged to the severe category. Proctitis was predominant in ulcerative colitis, while colonic involvement was predominant in Crohn’s disease (Table-I).

**Table-I:** Demographic and Clinical Characteristics of the Participants.

Parameter	Values	p-value
***Age (years), Mean±SD***		
Control (n=60)	36.7±3.3	
IBD (n=137)	36.4±12.2	0.864
***Sex, n (%)***		
Control - Male/Female	40 (66.7%)/20 (33.3%)	
IBD - Male/Female	90 (65.7%)/47 (34.3%)	0.853
***Disease Type Distribution, n (%)***		
Control	60 (30.5%)	
Ulcerative Colitis	90 (45.7%)	
Crohn’s Disease	40 (20.3%)	
***Disease Severity (IBD), n (%)***		
Mild	10 (7.7%)	
Moderate	60 (46.2%)	
Severe	60 (46.2%)	
***UC Localization Distribution (n=90)***		
Proctitis	40 (44.4%)	
Pancolitis	30 (33.3%)	
Left-sided Colitis	10 (11.1%)	
Extensive Colitis	10 (11.1%)	
***CD Localization Distribution (n=40)***		
Colonic	27 (67.5%)	
Ileocolonic	11 (27.5%)	
Ileal	2 (5.0%)	

***Notes:*** Mean: Mean value; SD: Standard deviation;

IBD: Inflammatory bowel disease;

UC: Ulcerative colitis;

CD = Crohn’s disease.

Nickel showed the most significant difference from controls, with IBD values approximately twice those of controls (1.19±1.04 vs. 0.51±0.39 µg/L; median 1.2 [IQR 0.5–1.6] vs. 0.4 [0.2–0.7] µg/L; p<0.001). Iron deficiency was observed in 54.0% of patients with IBD compared to 16.7% of healthy participants (p<0.001); ferritin (36.3±42.1 vs. 59.6±27.8 ng/mL; p<0.001) and zinc (91.0±16.8 vs. 100.1±16.3 µg/dL; p<0.001) levels were also lower. Selenium levels were comparable between groups (p=0.498), whereas patients showed lower vitamin D levels (30.9±10.0 vs. 33.8±11.6 ng/mL; p=0.025) (Table-II).

Although Pb, Cd, Hg, As, and Sn were also measured by ICP-MS, their concentrations remained below the limit of detection in the majority of samples and showed no statistically significant differences between groups; therefore, only nickel and aluminum data are reported.

Patients with Crohn’s disease had higher aluminum levels than those with ulcerative colitis (11.6±9.4 vs. 2.9±2.3 µg/L; p<0.001). Disease severity was associated with decreased selenium, vitamin D, and zinc concentrations (all p<0.001). In the mild severity subgroup (n=10), the mean nickel level was 4.0±0.6 µg/L; however, the median [IQR] was 1.1 [0.8–1.5] µg/L, indicating that this elevated mean was driven by a single outlier and that the central tendency was comparable to the moderate (median 0.8 [0.5–1.2] µg/L) and severe (median 0.9 [0.5–1.4] µg/L) subgroups. Aluminum strongly correlated with CRP (r=0.69, p<0.001); nickel showed a moderate correlation with calprotectin (r=0.56, p<0.001); whereas zinc (r=−0.42, p<0.001), selenium (r=−0.27, p<0.01) and vitamin D (r=−0.28, p<0.01) were negatively correlated with inflammatory markers. Proctitis and pancolitis showed the highest nickel and aluminum levels (p<0.001 for both) (Tables-II, III).

**Table-II:** Comparison of Heavy Metals and Trace Elements Between Groups and Subgroups.

Panel-A: Comparison Between Control and IBD Groups.			
Parameter (unit)	Control (n=60) Mean±SD [Median, IQR]	IBD (n=137) Mean±SD [Median, IQR]	p-value
***Heavy Metals***			
Nickel (µg/L)	0.51±0.39 [0.4, 0.2–0.7]	1.19±1.04 [1.2, 0.5–1.6]	<0.001***
Aluminum (µg/L)	5.54±3.59 [4.9, 3.0–7.5]	5.58±6.84 [3.5, 1.8–7.2]	0.045*
***Trace Elements***			
Iron (µg/dL)	82.4±35.4 [81.5, 58–105]	59.7±54.7 [33.0, 18–80]	<0.001***
Zinc (µg/dL)	100.1±16.3 [104.0, 90–112]	91.0±16.8 [88.0, 80–102]	<0.001***
Selenium (µg/L)	80.6±64.6 [70.5, 45–105]	79.8±49.8 [67.0, 50–95]	0.498
Vitamin D (ng/mL)	33.8±11.6 [30.0, 25–41]	30.9±10.0 [26.5, 23–37]	0.025*
Ferritin (ng/mL)	59.6±27.8 [45.0, 38–78]	36.3±42.1 [22.0, 12–45]	<0.001***

**Table:** Panel-A (continued): Categorical Distributions.

Parameter	Category	Control n (%)	IBD n (%)	p-value
Iron (µg/dL)	Low (<50)	10 (16.7%)	74 (54.0%)	<0.001***
	Normal (50–170)	50 (83.3%)	50 (36.5%)	
	High (>170)	0 (0.0%)	6 (4.4%)	
Nickel (µg/L)	Normal (0–1)	50 (83.3%)	62 (45.3%)	<0.001***
	High (>1)	10 (16.7%)	68 (49.6%)	

**Table:** Panel-B: IBD Subgroups and Disease Severity.

Parameter (unit)	Control (n=60)	UC (n=90)	CD (n=40)	Mild (n=10)	Moderate (n=60)	Severe (n=60)	p-value (type / severity)
***Heavy Metals***							
Nickel (µg/L) — Mean±SD	0.5±0.4	1.2±1.2	1.1±0.8	4.0±0.6†	0.9±0.6	1.0±0.7	<0.001*** / <0.001***
Nickel (µg/L) — Median [IQR]	0.4 [0.2–0.7]	0.9 [0.4–1.6]	1.0 [0.5–1.5]	1.1 [0.8–1.5]†	0.8 [0.5–1.2]	0.9 [0.5–1.4]	
Aluminum (µg/L) — Mean±SD	5.5±3.6	2.9±2.3	11.6±9.4	3.3±0.7	2.9±2.7	8.6±8.8	<0.001*** / <0.001***
Aluminum (µg/L) — Median [IQR]	4.9 [3.0–7.5]	2.4 [1.2–4.0]	9.0 [4.5–16.0]	3.2 [2.7–3.8]	2.1 [1.0–4.0]	6.0 [2.5–12.0]	
***Trace Elements***							
Selenium (µg/L) — Mean±SD	80.6±64.6	80.2±57.8	78.9±24.2	137.8±8.0	83.1±66.6	66.8±15.1	0.472 / <0.001***
Selenium (µg/L) — Median [IQR]	70.5 [45–105]	68 [42–100]	76 [60–95]	138 [132–143]	70 [40–110]	65 [55–78]	
Vitamin D (ng/mL) — Mean±SD	33.8±11.6	30.5±10.5	31 .9±9.0	49.8±1.4	29.6±10.1	29.1±7.3	0.047* / <0.001***
Vitamin D (ng/mL) — Median [IQR]	30.0 [25–41]	28 [22–37]	30 [25–38]	50 [49–51]	27 [22–36]	28 [24–34]	
Zinc (µg/dL) — Mean±SD	100.1±16.3	90.7±16.0	91.8±18.6	114.5±4.2	92.7±12.6	85.4±18.1	0.003** / <0.001***
Zinc (µg/dL) — Median [IQR]	104.0 [90–112]	88 [78–102]	90 [78–105]	114 [111–118]	92 [83–102]	84 [72–98]	

**Table:** Panel-C: Selenium Categorical Distribution by Disease Severity.

Category	Mild (n=10)	Moderate (n=60)	Severe (n=60)	p-value
Low (<70 µg/L)	0 (0.0%)	29 (48.3%)	40 (66.7%)	<0.001***
Normal (70–150 µg/L)	10 (100.0%)	30 (50.0%)	20 (33.3%)	
High (>150 µg/L)	0 (0.0%)	1 (1.7%)	0 (0.0%)	

***Notes:*** The Mann–Whitney U test (Panel A) and Kruskal–Wallis test with post-hoc Dunn test (Panels B, C) were used for continuous variables, and the Chi-square test was used for categorical variables. Continuous variables are presented as Mean±SD and Median [IQR]. †One outlier was identified in the mild severity subgroup (n=10) for nickel; both Mean±SD and Median [IQR] are reported to address the skewed distribution. IBD: Inflammatory bowel disease; UC: Ulcerative colitis; CD: Crohn’s disease; SD: Standard deviation; IQR: Interquartile range. *p<0.05, **p<0.01,

*** p<0.001.

**Fig.1:**
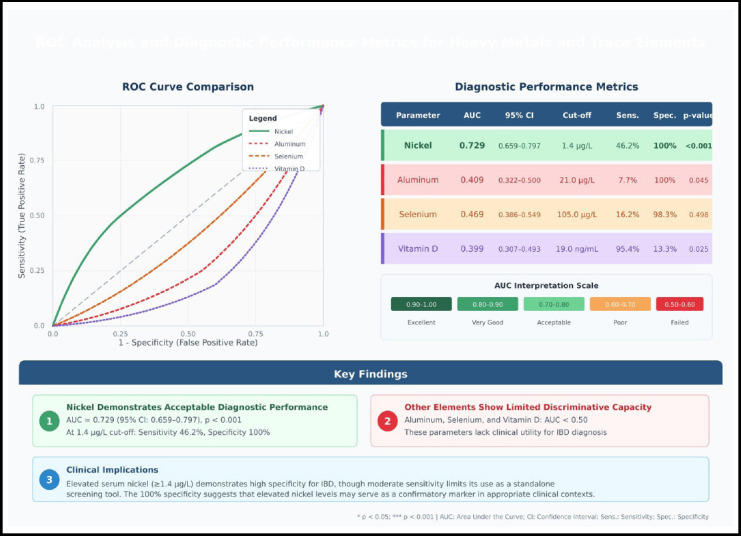
ROC analysis and diagnostic performance of heavy metals and trace elements in IBD. ROC: Receiver Operating Characteristic; AUC: Area Under the Curve; CI: Confidence Interval; IBD: Inflammatory Bowel Disease.

In multivariate analysis, high nickel levels increased the likelihood of IBD approximately seven-fold (OR=7.36, 95% CI: 3.23–16.75, p<0.001). Low iron levels also showed a significant association with the disease (OR=0.98, 95% CI: 0.97–0.99, p=0.002). Aluminum unexpectedly appeared protective (OR=0.90, 95% CI: 0.84–0.97, p=0.007); this finding should be interpreted with caution. The independent effects of selenium, vitamin D, and zinc concentrations were not significant (all p>0.05). In the ROC analysis, specificity reached 100% at a cut-off value of 1.4 µg/L for nickel, but sensitivity remained moderate. Aluminum, selenium, and vitamin D levels showed inadequate diagnostic performance (Table-III, Fig.1).

**Table-III:** Correlation Analysis, Disease Localization, and Multivariate Regression Results

Panel-A: Correlation with Inflammatory Markers			
Parameter	CRP	ESR	Calprotectin
Nickel (µg/L)	–	–	r=0.56***
Aluminum (µg/L)	r=0.69***	r=0.35***	r=0.27**
Selenium (µg/L)	r=−0.27**	r=−0.52***	–
Vitamin D (ng/mL)	–	r=−0.28**	–
Zinc (µg/dL)	r=−0.42***	r=−0.65***	r=−0.43***
Iron (µg/dL)	r=−0.22*	r=−0.27**	–

**Table:** Panel-B: Nickel and Aluminum Levels by UC Localization

Localization	Nickel (µg/L)	Aluminum (µg/L)
Proctitis	1.8±1.4	2.7±2.4
Left-sided Colitis	0.3±0.1	0.9±1.0
Pancolitis	1.2±0.4	4.5±1.6
Extensive Colitis	0.2±0.1	1.0±1.1
p-value	<0.001***	<0.001***

**Table:** Panel-C: Multivariate Logistic Regression Analysis

Variable	Beta	OR	95% CI	p-value
Nickel (µg/L)	1.995	7.36	3.23–16.75	<0.001***
Aluminum (µg/L)	−0.103	0.90	0.84–0.97	0.007**
Iron (µg/dL)	−0.018	0.98	0.97–0.99	0.002**
Selenium (µg/L)	−0.001	1.00	0.99–1.01	0.764
Vitamin D (ng/mL)	−0.021	0.98	0.94–1.02	0.289
Zinc (µg/dL)	−0.020	0.98	0.95–1.01	0.210
Age	−0.003	1.00	0.95–1.04	0.894
Sex (Male)	−0.224	0.80	0.31–2.08	0.645

***Notes:*** Panel A shows Spearman correlation coefficients; only significant correlations are displayed. Panel B presents Kruskal–Wallis test results for UC localization. Panel C shows multivariate logistic regression adjusted for age and sex. CRP: C-reactive protein; ESR: Erythrocyte sedimentation rate; IBD: Inflammatory bowel disease; UC: Ulcerative colitis; OR: Odds ratio; CI: Confidence interval. *p<0.05,

** p<0.01,

*** p<0.001.

## DISCUSSION

This study found that patients with IBD had lower antioxidant trace element levels, while heavy metal concentrations were elevated. With disease progression, nickel levels increased relative to iron and zinc, which was associated with more pronounced nutritional imbalances.

Patients with IBD had serum nickel levels approximately twice those of controls, with the highest levels observed in patients with ulcerative colitis presenting as proctitis. Research shows that patients with Crohn’s disease had higher nickel levels than patients with ulcerative colitis, possibly reflecting differences in population characteristics and disease patterns.^6^ It was also shown that people who consumed too much nickel developed lower numbers of *Lactobacillus* and *Bifidobacterium* but their *Escherichia coli* numbers remained stable which contributed to systemic inflammation and dysbiosis.^12^ Nickel nanoparticles damage keratinocyte tight junctions, with higher nickel exposure dramatically increasing the likelihood of developing IBD.^13^ Another study found that nickel levels increase only after initial IBD onset.^14^

In the present study, ulcerative colitis predominantly involved the rectum, with proctitis observed in 44.4% of patients, while pancolitis was identified in 33.3% and both left-sided and extensive colitis in 11.1%. This distribution differs markedly from a retrospective cohort study from Pakistan, in which extensive colitis (31.5%) and left-sided colitis (27.9%) were the most common patterns, whereas ulcerative proctitis accounted for only 18.2% of cases.^15^ Such regional differences may reflect variations in genetic susceptibility, environmental exposures, or dietary patterns, emphasizing the importance of evaluating environmental risk factors such as heavy metals in a population-specific context.

Patients with moderate IBD or those with anaemia frequently exhibit iron deficiency.^16^ A report established ferritin and hepcidin as diagnostic tools for European Crohn’s and Colitis Organization-guided diagnosis.^17^ Iron deficiency developed in 33.9% of non-anaemic patients with IBD who were women without mucosal healing,^18^ indicating immediate treatment with iron supplements. Zinc deficiency was observed in 54% of patients with Crohn’s disease and 41% of patients with ulcerative colitis.^19^ The blood zinc levels of patients showed a protective effect against the onset of ulcerative colitis (OR = 0.91).^20^ The treatment of Hirschsprung’s disease patients with zinc supplements resulted in a substantial reduction of enterocolitis occurrence from 40% to 12%.^21^ Hence, zinc supplements are indicated for patients with severe IBD.

The present study established that aluminum levels strongly correlated with those of CRP (r = 0.69), and patients with Crohn’s disease had higher aluminum levels than those with ulcerative colitis. Our multivariate analysis showed aluminum to be a protective factor (OR = 0.90), indicating that aluminum does not function as an independent risk factor. Aluminum exposure has been shown to trigger TNF-α and IL-17A production in Crohn’s disease but not in healthy tissue specimens.^22^ The selenium levels in patients matched those of controls (p = 0.498) but they decreased with disease progression. Selenium deficiency develops in patients with Crohn’s disease and ulcerative colitis and reaches its highest levels during symptomatic phases.^23^

To the best of our knowledge, this is among the first studies to simultaneously evaluate heavy metals and antioxidant trace elements in newly diagnosed IBD patients in relation to disease type, severity, and anatomical localization. The strong independent association of nickel with IBD and the high specificity of the proposed cut-off in ROC analysis suggest its potential utility as a candidate biomarker for risk stratification, while the progressive decline of zinc, selenium, and vitamin D with worsening severity supports early identification of micronutrient deficits and timely supplementation in routine IBD management.

### Strengths of this study:

The strengths of this study include a well-characterized cohort of newly diagnosed IBD patients, simultaneous ICP-MS quantification of multiple elements in a single calibrated laboratory with blinded personnel, and the integration of disease type, severity (Mayo and CDAI), and anatomical localization in the analysis. Reporting both mean ± SD and median [IQR] further enhances transparency in subgroups with skewed distributions. Mechanistically, nickel-induced dysbiosis and tight junction disruption may amplify mucosal permeability and chronic immune activation, while concomitant depletion of antioxidant trace elements likely impairs cellular defense against oxidative stress, creating a self-sustaining inflammatory loop.

### Limitations

The retrospective, single-center cross-sectional design precludes causal inference and limits generalizability. Potential confounders — including dietary habits, occupational heavy metal exposure, smoking status, drinking water source, and concomitant medications such as multivitamins or antacids — could not be systematically controlled. The mild severity subgroup was small (n=10), limiting statistical power; reporting of both mean ± SD and median [IQR] partially mitigates this concern. Single time-point measurement precludes assessment of temporal changes. Finally, although Pb, Cd, Hg, As, and Sn were measured by ICP-MS, their concentrations remained below the limit of detection in the majority of samples and could not be meaningfully evaluated.

## CONCLUSION

This study showed that elevated serum nickel and iron deficiency are associated with the presence and severity of IBD, while zinc, selenium, and vitamin D progressively decline with worsening disease severity. Nickel emerged as an independent factor associated with IBD and may serve as a candidate biomarker for risk stratification, whereas aluminum appears to reflect systemic inflammation rather than act as a causal contributor. These findings support the integration of heavy metal and trace element profiling into the clinical evaluation of newly diagnosed IBD patients and highlight the need for prospective, multicenter studies to validate these biomarkers.

### Recommendations

Future prospective, multicenter, longitudinal studies are needed to confirm the causal role of nickel and to assess whether serial monitoring predicts relapse or treatment response, alongside randomized trials evaluating targeted micronutrient supplementation in IBD.

### Use of Artificial Intelligence:

The authors declare that no artificial intelligence (AI) tools or large language models (such as ChatGPT) were used in the writing, drafting, or editing of this manuscript. All content was generated solely by the listed authors.

### Author’s Contribution:

**EA:** Conception, design, statistical analysis, editing; responsible for research integrity.

**EA, MSA & GU:** Data collection and manuscript writing.

**EA & GU:** Literature search, critical Review.

All authors have read and approved the final version of the manuscript.
